# *Lactobacillus plantarum* Surface-Displayed ASFV (p14.5) Can Stimulate Immune Responses in Mice

**DOI:** 10.3390/vaccines10030355

**Published:** 2022-02-24

**Authors:** Quntao Huang, Tianming Niu, Boshi Zou, Junhong Wang, Junhong Xin, Hui Niu, Nan Li, Yuxin Jiang, Junfu Bao, Di Zhang, Xize Feng, Tingting Sun, Xin Wang, Kaidian Yang, Ying Wang, Guilian Yang, Dandan Zhao, Chunfeng Wang

**Affiliations:** 1 College of Veterinary Medicine, Jilin Agricultural University, Changchun 130118, China; huangquntao97@gmail.com (Q.H.); 18686401118@163.com (T.N.); ssymantaci@gmail.com (B.Z.); 15754365128@163.com (J.W.); a18543127250@163.com (J.X.); niuhuinh0227@163.com (H.N.); lnanlnln@163.com (N.L.); jyx17390928094@163.com (Y.J.); junfubao20200422@126.com (J.B.); zdi632933@gmail.com (D.Z.); jlaufxz@126.com (X.F.); sun3412984149@163.com (T.S.); jinjinjin202112@163.com (X.W.); jh234789@163.com (K.Y.); wangying202106@163.com (Y.W.); 2 Jilin Provincial Engineering Research Center of Animal Probiotics, Jilin Provincial Key Laboratory of Animal Microecology and Healthy Breeding, Jilin Agricultural University, Changchun 130118, China; 3 Key Laboratory of Animal Production and Product Quality Safety of Ministry of Education, Jilin Agricultural University, Changchun 130118, China

**Keywords:** African Swine Fever Virus (ASFV), p14.5, CTA1-DD, IL-33, *L. plantarum*, immune evaluation

## Abstract

African Swine Fever Virus (ASFV) has spread worldwide, and the lack of vaccines severely negatively impacts the pig industry. In this study, the p14.5 protein encoded by ASFV was used as the antigen, and the p14.5 gene was expressed in vitro using the *Lactobacillus* expression system. Three new functionally recombinant *Lactobacillus plantarum* (*L. plantarum*) were constructed and the expressions of the p14.5 protein, p14.5-IL-33-Mus fusion protein and CTA1-p14.5-D-D fusion protein were successfully detected using Western blot analysis. After oral immunization of SPF mice with recombinant *L. plantarum*, flow cytometry and ELISA were performed to detect the differentiation and maturity of T lymphocytes, B lymphocytes and DCs of the mice, which were higher than those of the control group. Specific antibodies were produced. The immunogenicity of the adjuvant group was stronger than that of the single antigen group, and the IL-33 adjuvant effect was stronger than that of the CTA1-DD adjuvant.

## 1. Introduction

African Swine Fever (ASF) is an acute, febrile and highly contagious pig disease caused by the African Swine Fever Virus (ASFV). Clinical symptoms and pathological changes of ASF are similar to those of classic swine fever, and it is easily misdiagnosed. ASF specifically manifests as high fever, skin congestion, abortion, edema and organ bleeding. Its mortality rate is as high as 100% [1]. The causative agent of ASF, ASF virus (ASFV), is a double-stranded DNA virus, the sole member of the Asfarviridae family [2].

To date, no safe and efficacious treatment or vaccine against ASF is available. Nevertheless, there are several reports of protection elicited by experimental vaccines based on the subunit vaccine ASFV [3]. The entire genome of ASFV contains 167 open reading frames (ORFs), which can encode from 150–200 proteins and approximately 50 of them are structural proteins [4], including the p14.5 protein. As a late-expressing protein, p14.5 is a key virulence factor of ASFV. It can bind interferon regulatory factor 3 (IRF3) to block its recruitment and plays an important role in inhibiting IRF3 phosphorylation and interferon production. The p14.5 protein can cause a humoral response and is synthesized in the late stage of infection and is located in the virus factory. It is a protein necessary for the transfer of virus particles from the virus factory to the plasma membrane and can combine with the P72 protein to form the virus capsid.

CTA1-DD is an artificial adjuvant composed of the enzymatically active CTA1 subunit of cholera toxin and the D domain dimer of the Staphylococcus aureus protein A [5]. This molecule has previously been demonstrated to be non-toxic in mice and non-human primates and can be used as a safe adjuvant. It can stimulate robust and balanced CD4^+^ T cell responses, greatly enhancing specific antibody production. After systemic and mucosal immune responses, follicular dendritic cells (FDCs) in peripheral lymph nodes can promote germinal center B cells and Tfh responses [6]. IL-33 is a multifunctional protein discovered in 2005. It is secreted during cell damage and plays a role in regulating immunity [7]. IL-33 can directly participate in DC responses to stimulate the differentiation of naive T cells into Th2 or helper T cells [8]. During viral infection, IL-33 can help clear a virus by promoting the expansion of NK and NKT cells and enhancing Th1 and CD8^+^T cell responses [9].

*Lactobacillus* species are an important physiological flora essential to the human body. They are widely found in the human gut, regulating human intestinal health, and are directly related to health and longevity. *Lactobacillus* has obvious advantages as a carrier by presenting biomolecules to the gastrointestinal tract for the prevention and treatment of diseases. It can also stimulate the body to produce an effective and long-term immune response. The S-layer protein derived from *Lactobacillus* is a single-molecule crystal structural protein on the outermost surface of cells. It is considered to form the outermost structure of the cell membrane in many archaea [10], displaying foreign proteins on its surface. The S-layer protein not only has the ability to anchor and express foreign proteins but also has adhesive properties. It can be used as an adjuvant to stimulate the body’s immune responses [11]. In this study, to anchor the expressed protein in the S layer, we used the vector pLP-S (pLp_1261Inv derivative, in which the lp_1261 and Inv fusion gene was replaced with the SP-linker-S_anchoring sequence of the SlpA gene containing MCS1), and p14.5, p14.5-IL-33-Mus and CTA1-p14.5-D-D gene sequences were inserted to construct three new plasmids. The plasmids were subsequently transformed into *Lactobacillus plantarum* NC8 to establish three recombinant *L. plantarum*. *L. plantarum* can colonize the mouse intestinal tract and express the p14.5 protein and p14.5-IL-33 and CTA1-p14.5-D-D fusion proteins in the intestinal environment. The ELISA experiment proved that the host body can recognize the p14.5 protein and the p14.5-IL-33 and CTA1-p14.5-D-D fusion proteins and produce specific antibodies.

Preliminary animal experiments were carried out on mice. The results showed that feeding recombinant *L. plantarum* had an effect on improving the immunity of mice, promoting the differentiation and maturation of T and B lymphocytes and DC cells, and stimulating T cells to produce relatively high amounts of cytokines.

Furthermore, CTA1-DD exhibited an immune adjuvant effect, which was superior to IL-33. The present study may provide support for the development of new *L. plantarum* vaccines and immune adjuvants.

## 2. Materials and Methods

### 2.1. Animals and Ethics Statement

The animals used in this study were purchased from HFK Bioscience Co., Ltd. (Beijing, China). Pathogen-free female C57BL/6 mice aged 6–8 weeks were raised in SPF rooms. The animal experiments met the requirements of the Animal Management and were approved by the Ethics Committee of Jilin Agricultural University.

### 2.2. Construction of Recombinant L. plantarum

The base sequences of the ASFV E120R gene (gene number: 41901181) and IL-33-Mus gene (gene number: AY905582.1) were obtained from NCBI, and the base sequences of the CTA1 gene and DD gene (gene number: OL547739) were obtained from the literature and optimized and synthesized by Nanjing Genscript synthesis. The p14.5, p14.5-IL-33-Mus and CTA1-p14.5-D-D genes were cloned into the pLP-S vector. The constructed plasmid was transformed into *L. plantarum* NC8 (CCUG61730). Three recombinant *L. plantarum* including NC8-pLP-S-p14.5, NC8-pLP-S-p14.5-IL-33-Mus and NC8-pLP-S-CTA1-p14.5-D-D were generated. The bacteria were sequenced and identified by Comate Bioscience Co., Ltd. (Changchun, China).

### 2.3. Preparation of p14.5 Protein

The p14.5 gene sequence was inserted into the pET28a expression vector to construct the plasmid pET-28a-p14.5. Then the plasmid was transformed into *E. coli* competent BL21 cells to obtain recombinant *E. coli* BL21-pET-28a-p14.5. IPTG (100 mM) was used to induce protein expression. Cells were sonicated and proteins were collected from the inclusion bodies. The p14.5 protein was recovered after purification.

### 2.4. Western Blot

The preserved bacteria NC8-pLP-S-p14.5, NC8-pLP-S-p14.5-IL-33-Mus and NC8-pLP-S-CTA1-p14.5-D-D were transferred from −80 °C to the refrigerator. Thereafter, the bacteria were inoculated into 5 mL of MRS liquid containing Erm (5 μg/mL) and cultured overnight. The next day, they were transferred to 200 mL of MRS liquid and Erm (5 μg/mL) was added. After culturing at a 37 °C anaerobic workstation with a OD500 value of 0.3, 125 μL (50 ng/mL) of sakacin P inducer (SppIP) was added and the culture was induced for 8 h. After centrifugation, the precipitate was ultrasonically broken, and Western blot was performed after processing the sample. After separation by SDS-PAGE (10% acrylamide), the bacterial proteins were transferred onto a nitrocellulose membrane and incubated with antibodies conjugated with horseradish peroxidase (HRP) (Cell Signaling Technology, Danvers, MA, USA). After washing, proteins were visualized by enhanced chemiluminescence (Cell Signaling Technology, Danvers, MA, USA) on an Amersham Imager (General Electric Company, Boston, MA, USA).

### 2.5. Immunization

Fifty mice were randomly divided into five groups, with ten mice in each group. The mice were orally administered with 200 μL of NC8-pLP-S, NC8-pLP-S-p14.5, NC8-pLP-S-p14.5-IL-33-Mus or NC8-pLP-S-CTA1-p14.5-D-D with 1 × 10^9^ CFU colony-forming units. The control group was given 0.9% normal saline (200 μL) by the same method. The mice were first immunized on the 1st, 2nd and 3rd days, then received booster immunization on the 10th, 11th and 12th days, and finally boosted again on the 21st, 22nd and 23rd days. After the 1st immunization and the 3rd immunization, three mice from each group were randomly selected for flow cytometry analysis. The feces of each mouse were collected for ELISA analysis on the 0, 9th, 19th and 29th days.

### 2.6. Single Cell Suspension Preparation

On an ultra-clean table, autoclaved ophthalmic scissors and ophthalmic forceps were used to peel off the mesenteric lymph nodes (MLNs), Peyer’s collective lymph nodes (PPs) and the spleen. The excess fat was removed and placed on ice. A folded 200 mesh sterile filter was placed in a sterile small plate, and 1 mL of RPMI-1640 complete medium was added. The spleen was put into the strainer and gently grounded with the end of a sterile 1 mL syringe. Then, the liquid was aspirated into a 1.5 mL EP tube and centrifuged in a pre-cooled centrifuge at 4 °C, 2000× *g* rpm for 5 min. Then, the supernatant was discarded, 0.5 mL of red blood cell lysate buffer Beyotime Biotechnology was added, and the sample was lysed on ice for 3 min. Thereafter, 0.5 mL of PBS was added, and the sample was mixed for 2 min, centrifuged at 2000× *g* rpm, 4 °C for 5 min, and the supernatant was discarded. The pellet was washed with 1 mL PBS once, the supernatant was discarded, and 1 mL of complete medium was added. The cells were counted on a cell counting plate after diluting 100-fold. MLN and PP knots were similarly treated but there was no need to lyse them with red blood cell lysate buffer. After treatment, they were diluted 20-fold and counted.

### 2.7. Flow Cytometry

B220, IgA, CD11C and CD80/CD86 antibodies were used to stain PPs, and CD3, 4, 8, IL-4 and IFN-γ antibodies were used to stain single-cell suspensions of the spleen (SP) and MLNs. All antibodies were purchased from BD Biosciences (New York, NY, USA). We transferred 10 μL of anti-B220 antibody to a tube containing 1.5 × 10^6^ cells, mixed the solution well and stained the cells for 30 min at 4 °C in the dark. Then, 1 mL of phosphate-buffered saline (PBS) was added to make a cell suspension, centrifuged at 4 °C, 2000× *g* rpm for 5 min, and the supernatant was discarded. The above steps were repeated. Cells were then fixed and permeabilized, centrifuged twice, and stained with 10 μL of anti-IgA antibody for 30 min at 4 °C, protecting from the light using the same procedure described above. Then, the inhibitor was added for 3 h. Cells were centrifuged twice and 10 μL of anti-CD3, anti-CD4, and anti-CD8 antibodies were added. Next, cells were fixed, permeabilized, centrifuged twice, followed by adding 10 μL of anti-IFN-γ antibody and mixing for 20 min at 4 °C in the dark. BD fluorescence-activated cells were sorted and analyzed by FACS using an LSRFortessa analyzer (BD Bioscience, USA). All data were analyzed using FlowJo 7.6 software.

### 2.8. Enzyme-Linked Immunosorbent Assay

The presence of antigens that specifically bound to IgA antibodies in serum and fecal supernatants was assessed by enzyme-linked immunosorbent assay (ELISA) as described previously, with some minor alterations.Briefly, a 96-well polystyrene microtiter plate was coated with 1 μg of p14.5 antigen in a carbonate–bicarbonate buffer (pH9.6) and incubated overnight at 4 °C. Ten wells were sealed with 150 μL of blocking solution (PBST containing 10% bovine serum albumin) and incubated at 37 °C for 1 h. The wells were washed three times with PBST. Diluted samples were added to the well, incubated at 37 °C for 2 h, and then washed three times with PBST. Subsequently, 100 μL of anti-mouse His conjugate (Cell Signaling Technology, Danvers, MA, USA) at a dilution of 1:5000 was added to all wells and incubated for 1 h. After washing four times with wash buffer, the plate was developed with 0.02% O-phenylenediamine and 0.015% H_2_O_2_ (Thermo Fisher, Shanghai, China) in substrate buffer (15 mM citrate buffer pH 5.6), and the reaction was stopped after 10 min incubation with 2N H_2_SO_4_. The absorbance was read at 450 nm. Finally, the termination solution was added to stop the reaction. OD value was determined by a spectrophotometer. The final titer was evaluated as the highest dilution, resulting in twice the absorbance of the sample background.

### 2.9. Statistical Analysis

All data came from at least three independent experiments and were expressed as mean ± SEM. GraphPad Prism 5.0 software was used to compare the differences. *p* < 0.05 was considered to represent a significant difference. Analysis of variance (ANOVA) with Tukey’s multiple comparison test was used to evaluate the significance.

## 3. Results

### 3.1. Construction of Plasmids and Expression of Target Genes In Vitro

**Figure 1:**
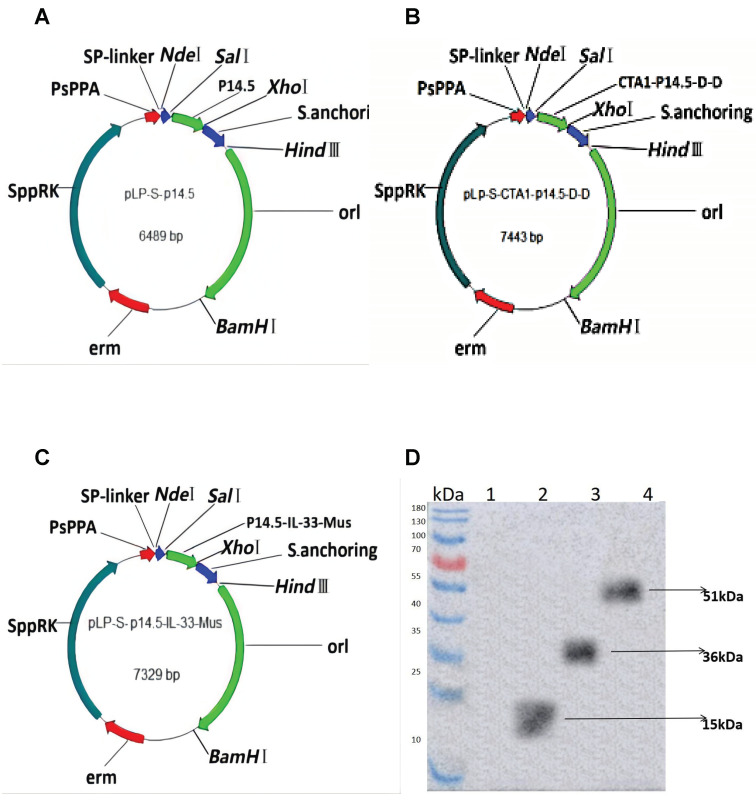
(**A**) A map of the plasmid NC8-pLP-S-p14.5,the plasmid size is 6489 bp; (**B**) A map of the plasmid NC8-pLP-S-p14.5-IL-33-Mus, the plasmid size is 7443 bp; (**C**) A map of the plasmid NC8-pLP-S-CTA1-p14.5-D-D, the plasmid size is 7329 bp; (**D**) The expressions of the recombinant *L. plantarum* ASFV p14.5 protein, p14.5-IL-21-Mus fusion protein and CTA1-p14.5-D-D fusion protein verified by Western blot, lane 1: NC8-pLP-S, lane 2:NC8-pLP-S-p14.5, lane 3: NC8-pLP-S-p14.5-IL-21-Mus and lane 4: NC8-pLP-S-CTA1-p14.5-D-D. The p14.5 molecular weight is 15 kDa, the p14.5-IL-33 molecular weight is 36 kDa and the CTA1-p14.5-D-D molecular weight is 51 kDa.

Three new functionally recombinant *L. plantarum* were successfully constructed (Figure 1A–C). The expressions of the p14.5 protein, the p14.5-IL-33 fusion protein and CTA1-p14.5-D-D protein were successfully detected using Western blot (with His-tag as the detection antigen) (Figure 1D). The p14.5 molecular weight was 15 kDa, the p14.5-IL-33 molecular weight was 36 kDa and the CTA1-p14.5-D-D molecular weight was 51 kDa. The results showed that the target band was consistent with the expected size, which proved that the recombinant *L. plantarum* successfully expressed the foreign protein.

### 3.2. Cellular Immune Responses Induced by Recombinant L. plantarum

**Figure 2:**
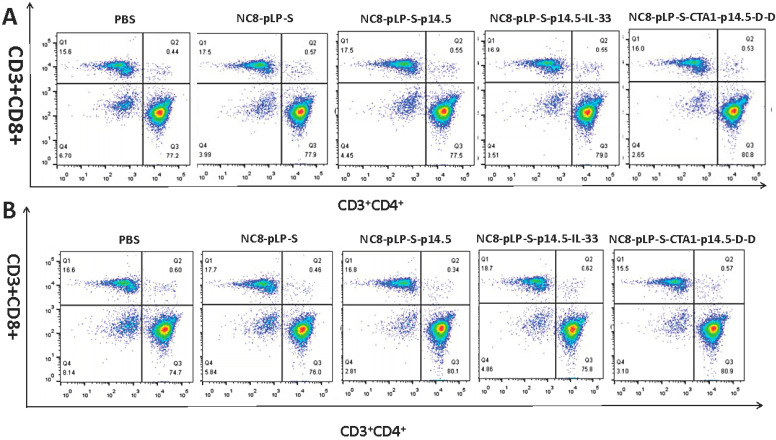
Flow cytometry to detect the degree of T cell differentiation. (**A**) Changes in the numbers of CD3^+^CD4^+^ and CD3^+^CD8^+^ T cells in the spleen; (**B**) Changes in the numbers of CD3^+^CD4^+^ and CD3^+^CD8^+^ T cells in the mesenteric lymph nodes. The degree of T cell differentiation in descending order is the CTA1-DD adjuvant group, IL-33 adjuvant group, single antigen group, empty vector group and control group.

**Figure 3:**
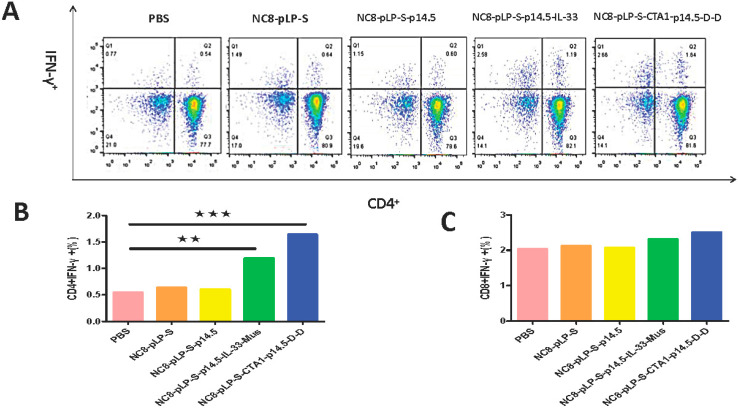
Flow cytometry to detect IFN-γ secretion. (**A**) Changes in CD4^+^IFN-γ^+^ in the spleen; (**B**) Statistical histogram of CD4^+^IFN-γ^+^ changes in spleen; (**C**) Statistical histogram of CD8^+^IFN-γ^+^ changes in spleen. The degree of B cell differentiation in descending order is the CTA1-DD adjuvant group, IL-33 adjuvant group, single antigen group, empty vector group and the control group. ** *p* < 0.01 and *** *p* < 0.001 compared with PBS group.

CD3, CD4 and CD8 are the surface markers of T lymphocytes, which can help to distinguish between cytotoxic and helper T cells. IFN-γ exhibits anti-viral, anti-tumor and immune regulation effects. It can also promote NK cell activity and antigen presentation and increase macrophage lysosomal activity. The results of flow cytometry showed that the number of CD3^+^CD8^+^ T cells and CD3^+^CD4^+^ T cells in the spleen and mesenteric lymph nodes increased significantly after the mice were orally administered with recombinant *L. plantarum* (Figure 2A,B), and the level of IFN-γ was significantly increased (Figure 3A–C). According to the degree of T cell differentiation from large to small, the sequence was the CTA1-DD adjuvant group, IL-33 adjuvant group, single antigen group, empty vector group and the control group. Comparing the two adjuvant groups, the CD3^+^ T cells in the CTA1-DD adjuvant group had a higher degree of differentiation towards the CD3^+^CD4^+^ cells, while the CD3^+^ T cells in the IL-33 adjuvant group had a higher degree of differentiation towards the CD3^+^CD8^+^ T cells. Therefore, recombinant *L. plantarum* improved mice immunity.

### 3.3. Mucosal Immune Responses Induced by Recombinant L. plantarum

**Figure 4:**
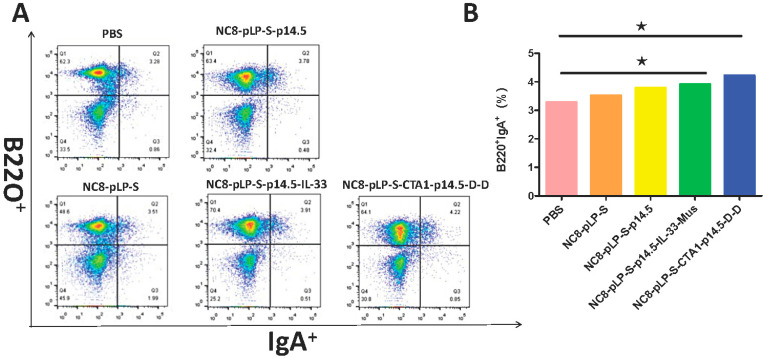
Flow cytometry to detect IgA secretion. (**A**) Changes in B220^+^IgA^+^ in mouse PPs; (**B**) Statistical histogram of B220^+^IgA^+^ changes in mouse PPs. The secretion of B220^+^IgA^+^ in descending order is the CTA1-DD adjuvant group, IL-33 adjuvant group, single antigen group, empty vector group and the control group. * *p* < 0.05 compared with PBS group.

**Figure 5:**
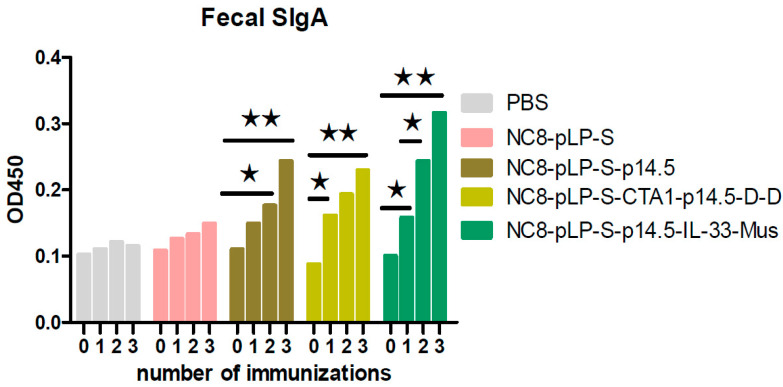
Detection of mouse feces using ELISA assay. Comparison of the increase in fecal IgA content in the five groups of mice after three immunizations. The secretion of SIgA in descending order is the CTA1-DD adjuvant group, IL-33 adjuvant group, single antigen group, empty vector group and the control group. * *p* < 0.05 and ** *p* < 0.01 compared with the SIgA before immunization.

Recombinant *L. plantarum* significantly improved the number of B220^+^IgA^+^ cells in PPs (Figure 4). The results showed that the degree of B cell differentiation in descending order was the CTA1-DD adjuvant group, IL-33 adjuvant group, single antigen group, empty vector group and the control group. The results of SIgA detection in mouse feces showed that with the increased number of immunizations with NC8-pLP-S-p14.5, NC8-pLP-S-p14.5-IL-33-Mus and NC8-pLP-S-CTA1-p14.5-D-D bacteria, the content of SIgA in the feces of mice increased gradually and significantly. After the third immunization, mice orally administered with NC8-pLP-S-p14.5, NC8-pLP-S-p14.5-IL-33-Mus and NC8-pLP-S-CTA1-p14.5-D-D bacteria showed significantly higher content of IgA in feces compared with mice orally administered with PBS, and there was no significant difference in the content of SIgA in mice feces between the PBS group and the empty carrier NC8-pLP-S group (Figure 5). The results showed that the recombinant *L. plantarum* effectively induced mucosal immunity in mice and produced sIgA at the same time.

## 4. Discussion

African swine fever has caused huge economic losses for the pig breeding industry worldwide. Therefore, research on a ASF subunit vaccine is of great significance [12]. *L. plantarum* is commonly used as a delivery vector to construct genetically engineered vaccines against viruses [13]. They can simultaneously activate the body’s cellular, humoral and mucosal immunity [14]. *L. plantarum* has immense potential as a mucosal vaccine vector. Yang et al. constructed a recombinant *Lactobacillus* NC8 expressing the influenza fusion genes *HA2* and *3M2e*. After the immunization of mice, as well as H9N2 and H1N1 challenge tests, the results showed that *L. plantarum* could provide effective protection, while reducing the extent of lung disease [15]. Huang and Jiang et al. successfully expressed the porcine epidemic diarrhea virus S protein and avian influenza virus H9N2 subtype HA2 protein with the pSIP409-pgsA’ anchored expression vector, achieving oral immunization of the engineered bacteria, which were found to have a moderate anti-coccidial effect on immunized chicks [16]. Anchoring the *L. plantarum* containing the HA2 protein and the adjuvant molecule LTB could significantly improve the anti-avian influenza virus ability of immunized mice. Chen et al. constructed a recombinant *L. plantarum* NC8 expressing influenza P54 and IL-21 fusion gene and evaluated the immune effect of NC8-pSIP409-pgsA’-p54-pIL-21 in a mouse model. The results of feeding mice recombinant L. plantarum showed that the levels of serum IgG and mucosal secreted IgA (SIgA), the number of CD4 and CD8 T cells, and the expression of IFN-γ in CD4 and CD8 T cells increased significantly, and lymphocyte proliferation occurred under stimulation with the ASFV p54 protein. In our study, the SIgA content in mice feces in the experimental group increased significantly after each immunization and reached the highest value after the third immunization, which was consistent with the mucosal immunity induced by NC8-pSIP409-pgsA’-p54-pIL-21.

Studies have proven that exogenous IL-33 can enhance the antiviral protection against influenza virus infection. Exogenous IL-33 induces the recruitment of dendritic cells, increases the secretion of pro-inflammatory cytokine IL-12 and promotes cytotoxic T cell responses in the local microenvironment [17]. There is extensive evidence that IL-33 has an anti-virus effect. Because IL-33-Mus and IL-33-Pig were highly homologous, this study used IL-33 as a potential mucosal adjuvant and provided a preliminary reference for future experiments with pigs. CTA1-DD, as a mucosal adjuvant for mucosal vaccines, could promote mucosal, humoral and cell-mediated immune responses, and the addition of CTA1-DD to a split vaccine provided 100% protection against lethal infection by the H3N2 virus [18]. In this study, we fused and expressed two different adjuvants with the p14.5 protein, respectively. The final result showed that the adjuvant effect of CTA1-DD was stronger than that of IL-33. This result may be related to various factors, and further experiments are still needed.

The flow cytometry experiment showed that the secretion of B220 and IgA, and the contents of CD11C^+^CD80 and CD11C^+^CD86 cells in the PP nodal lymphocytes of the mice in the recombinant *L. plantarum* group, were greater than those of the control group. The adjuvant could promote B cells to secrete specific antibodies and promote the differentiation of DC cells. The spleen and mesenteric lymph nodes of mice orally administered with recombinant *L. plantarum* had more CD3^+^CD4^+^ and CD3^+^CD8^+^ T cells than the control group. The adjuvant group had more T cells than the single antigen group, which proved that the recombinant *L. plantarum* promoted the differentiation of mouse T cells, and the adjuvant group had a better promoting effect. The expressions of CD4^+^IFN-γ and CD8^+^IFN-γ in the spleen and mesenteric lymph nodes of mice orally administered with recombinant *L. plantarum* increased significantly, and the increase in the adjuvant group was higher than those in the single antigen group. ELISA was used to detect the specific SIgA content in mice feces, which proved that the recombinant *L. plantarum* group stimulated the host to produce specific antibodies, and the secretion of the adjuvant group was higher than that of the single antigen group.

The above experimental results showed that the immunity of mice was significantly improved when orally administered with new functionally recombinant *L. plantarum*. Recombinant *L. plantarum* could improve the humoral, cellular and mucosal immunity of mice, providing a theoretical basis for the development of an oral ASFV vaccine. A mucosal vaccine may have an effect on blocking the ASFV infection through mucosa. This study provided preliminary guidance for future vaccine research.

## Data Availability

The raw data reported in this manuscript have been deposited in Jilin Agricultural University, Changchun, China.

## Abbreviations

ASFV	African Swine Fever Virus
*L. plantarum*	*Lactobacillus plantarum*
ASF	African Swine Fever
ORFs	Open reading frames
FDCs	Follicular dendritic cells
MLNs	Mesenteric lymph nodes
PPs	Pey’s collective lymph nodes
SP	Spleen
P14.5-IL-33-Mus	p14.5-IL-33-mouse
